# SeqEnhDL: sequence-based classification of cell type-specific enhancers using deep learning models

**DOI:** 10.1186/s13104-021-05518-7

**Published:** 2021-03-19

**Authors:** Yupeng Wang, Rosario B. Jaime-Lara, Abhrarup Roy, Ying Sun, Xinyue Liu, Paule V. Joseph

**Affiliations:** 1 BDX Research and Consulting LLC, Herndon, VA 20171 USA; 2 Division of Intramural Clinical and Biological Research (DICBR), National Institute on Alcohol Abuse and Alcoholism, National Institutes of Health, Bethesda, MD 20892 USA; 3 Division of Intramural Research, National Institute of Nursing Research, National Institutes of Health, Bethesda, MD 20892 USA

**Keywords:** Enhancer, Classification, Deep learning, DNA sequence, Cell type

## Abstract

**Objective:**

To address the challenge of computational identification of cell type-specific regulatory elements on a genome-wide scale.

**Results:**

We propose SeqEnhDL, a deep learning framework for classifying cell type-specific enhancers based on sequence features. DNA sequences of “strong enhancer” chromatin states in nine cell types from the ENCODE project were retrieved to build and test enhancer classifiers. For any DNA sequence, positional *k*-mer (*k* = 5, 7, 9 and 11) fold changes relative to randomly selected non-coding sequences across each nucleotide position were used as features for deep learning models. Three deep learning models were implemented, including multi-layer perceptron (MLP), Convolutional Neural Network (CNN) and Recurrent Neural Network (RNN). All models in SeqEnhDL outperform state-of-the-art enhancer classifiers (including gkm-SVM and DanQ) in distinguishing cell type-specific enhancers from randomly selected non-coding sequences. Moreover, SeqEnhDL can directly discriminate enhancers from different cell types, which has not been achieved by other enhancer classifiers. Our analysis suggests that both enhancers and their tissue-specificity can be accurately identified based on their sequence features. SeqEnhDL is publicly available at https://github.com/wyp1125/SeqEnhDL.

**Supplementary Information:**

The online version contains supplementary material available at 10.1186/s13104-021-05518-7.

## Introduction

Cell type-specific enhancers, *cis*-regulatory elements that up-regulate gene transcription in a cell type, play a key role in determining the regulatory landscape of the human genome [1]. Enhancers are commonly located in the introns and immediately upstream of their target genes’ transcription start site (TSS). They are also known to populate gene deserts [2], reside in introns of neighboring genes [3], and co-localize with coding exons [4]. Enhancer mutations are often associated with diseases [5–7]. Accurate prediction of enhancers from DNA sequences is the basis of assessing whether mutation(s) can disrupt an enhancer’s activity, a type of mechanism for genetic diseases.

Predicting enhancers based on transcription factor binding sites (TFBS) was proposed because TFBS tend to be conserved over vertebrate evolution [8–10]. However, there is uncertainty regarding the identification of TFBS from DNA sequences. To ameliorate this challenge, direct sequence features such as *k*-mers (i.e., nucleotide sequences with a specified length) were then introduced to model enhancer prediction [11, 12]. However, these early studies did not achieve high prediction accuracy nor were they able to distinguish enhancers of different cell types.

With the wide application of ChIP-seq technologies, enhancers were frequently profiled on a genome-wide scale [13]. The ENCODE project produced genome-wide profiles of various epigenetic marks for multiple human cell types [14]. By applying a hidden Markov model (i.e. ChromHMM) to these epigenetic marks, the human genome sequence has been binned into more than ten chromatin states, including enhancers [15, 16]. The “strong enhancer” state, shown to be associated with increased gene expression, provides genome-wide positioning of active enhancers specific to a cell type [15]. Although these datasets’ availability renders enhancers’ positioning unnecessary, enhancers’ sequence structures, especially their subtle differences among cell types, can help understand cell type-specific gene regulation and should be explored.

The proper generation of negative sequences influences the effectiveness of enhancer classifiers. Negative sequences should contain similar basic sequence features with enhancers such as length distributions, GC, and repeat contents [12, 17, 18]; otherwise, enhancer classifiers may learn different nucleotide compositions rather than occurrences of key DNA motifs. Although many studies reported sequence-based enhancer prediction, it is still unknown whether enhancers can be distinguished between different cell types or tissues based on sequences.

The sequence structures of enhancers may not be linear or additive. In fact, there could be complex grammar or semantics among different DNA elements of an enhancer [19, 20]. In recent years, deep learning technologies have gained greater popularity compared to conventional machine learning methods, and have been adapted in biomedical research to address complex research questions [21–29]. Thus, deep learning can be more powerful in classifying enhancers. In this study, we propose SeqEnhDL, a deep learning framework for the classification of cell type-specific enhancers based on sequence features. To include interdependency and sequence information in the features of a DNA sequence, SeqEnhDL uses positional *k*-mer fold changes across each nucleotide position as its features. The effectiveness and advantages of SeqEnhDL are demonstrated based on the chromatin state segmentation data of nine cell types from the ENCODE project [14].

## Main text

### Methods

#### Genome annotations

The sequences and transcripts of the human genome (hg19) were obtained from the UCSC genome browser. The “knowngene” dataset was used to guide masking exons. Chromatin state annotations of gm12878, H1hesc, hepg2, Hmec, Hsmm, Huvec, K562, Nhek, and Nhlf cell types generated by ChromHMM [30] were obtained from the ENCODE project (Broad version). The data included a total of 15 chromatin states. 4_Strong_Enhancer and 5_Strong_Enhancer states were used as enhancers in this study.

#### Detailed computational procedure

See Additional file 1: methods.

## Results

### The SeqEnhDL framework

**Fig. 1:**
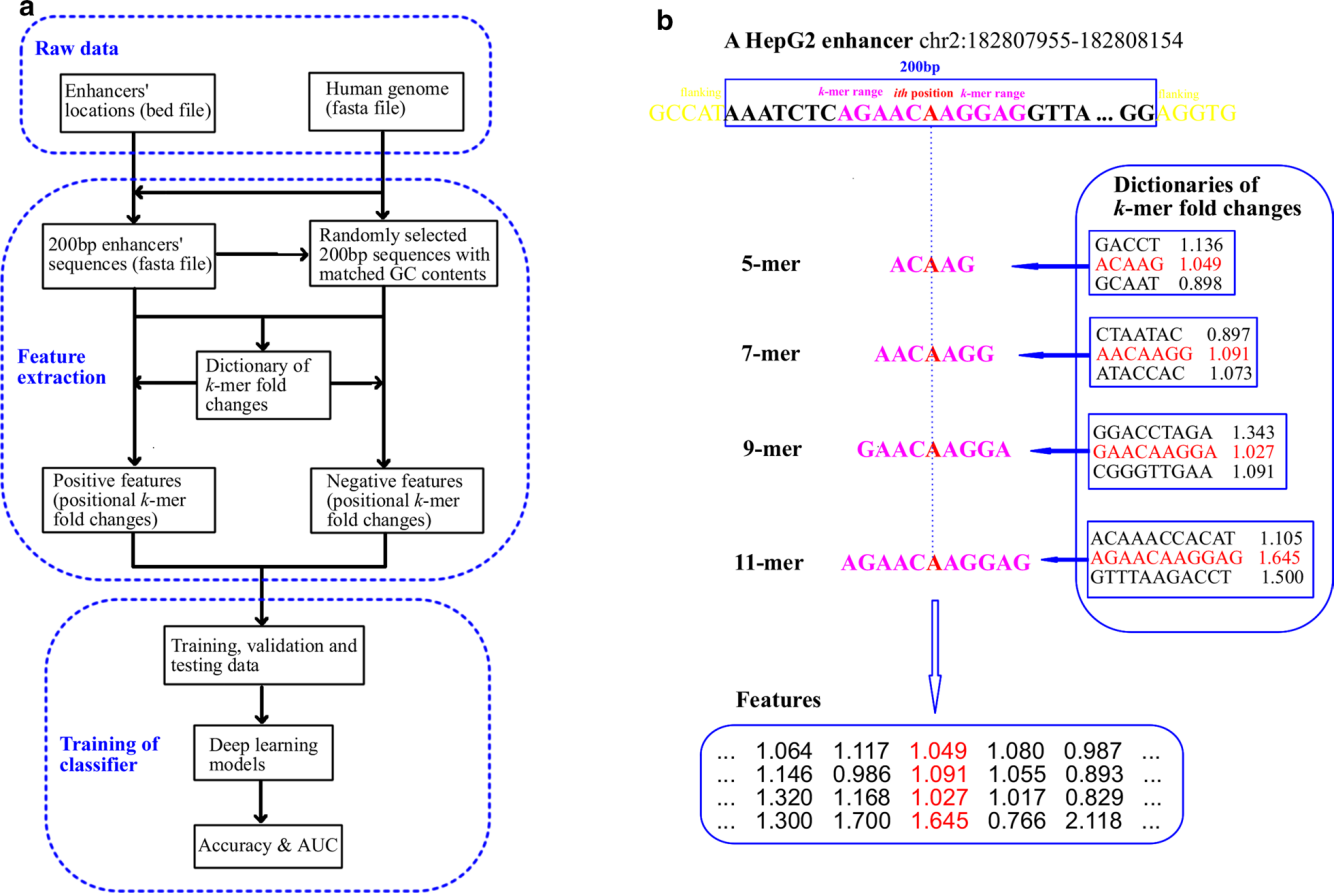
The SeqEnhDL approach. **a** Flowchart of the general SeqEnhDL procedure. A more detailed flowchart is available in the Additional file 1: Figure S1. **b** An intuitive example of the positional *k*-mer fold changes for sequence representation. The enhancer sequence at chr2: 182,807,955–182,808,154, with 5 bp flanking regions, is displayed. The example shows how to generate features for the 13th position (nucleotide “A”) among the 200 bp enhancer region. 5, 7, 9, and 11-mer centerred at the nucleotide “A” is extracted. Then, these k-mers are searched against dictionaries for their fold changes. Finally, the features at the 13th position are represented by the fold changes of its 5, 7, 9, and 11-mers

The SeqEnhDL framework is depicted in Fig. 1a. The framework started from “bed” files containing the chromosomal positions of a large number (e.g. > 1000) of enhancers for a cell type. The DNA sequences of enhancers were retrieved from the human genome where exon and repetitive sequences were masked. These DNA sequences were then divided into individual enhancers with a fixed length of 200 bp, which makes features more standardized and comparable. 200 bp is recommended because it corresponds with the resolution of a nucleosome and spacer region, though other lengths can be used. Enhancer sequences were used as the positive sequences. Control sequences for computing *k*-mer fold changes, and negative sequences for testing enhancer classifiers, were randomly selected from the genome where exon, repetitive and enhancer sequences were masked, according to the GC contents of enhancer sequences. *K*-mer (*k* = 5, 7, 9, and 11) fold changes between all enhancer and control sequences of a cell type were computed and used as dictionaries. To convert a DNA sequence to features, *k*-mer (*k* = 5, 7, 9, and 11) fold changes at each nucleotide position, referred to as positional *k*-mer fold changes, were generated according to the dictionaries of the cell type. Of note, we chose odd *k*-mers because fold changes of different *k*-mers can be aligned at their central nucleotide position. Then, features of each nucleotide position of a DNA sequence were concatenated, resulting in a 200 × 4 array of features for that DNA sequence. An intuitive example for the feature extraction process is shown in Fig. 1b. Thus, our feature extraction process retained interdependency and sequence information among the nucleotide positions of a DNA sequence.

Any dataset for building a deep learning enhancer classifier should be divided into training, validation, and testing data, in a cross-validation mode (e.g., 5-fold). Evaluation metrics were based on the average of each fold of cross-validation. Three deep learning models-MLP, CNN, and RNN, were built. Multilayer perceptrons are fully connected networks. CNN takes advantage of the hierarchical pattern in data and assembles more complex patterns using smaller and simpler patterns. RNN makes use of sequential information among features. Particularly, bidirectional long short-term memory (LSTM) RNN, which can learn long-term dependencies, was adopted. Because we adopted the positional *k*-mer fold changes, which utilize nucleotide position indexing, features at different positions (especially adjacent positions) could be interdependent. In addition, different *k*-mers [5, 7, 9, 11] at a nucleotide position represented the second dimension of features. The interdependency and sequence information of positional *k*-mer fold changes among nucleotide positions rationalize the use of CNN and RNN architectures.

### Evaluation of the performance of SeqEnhDL

**Fig. 2:**
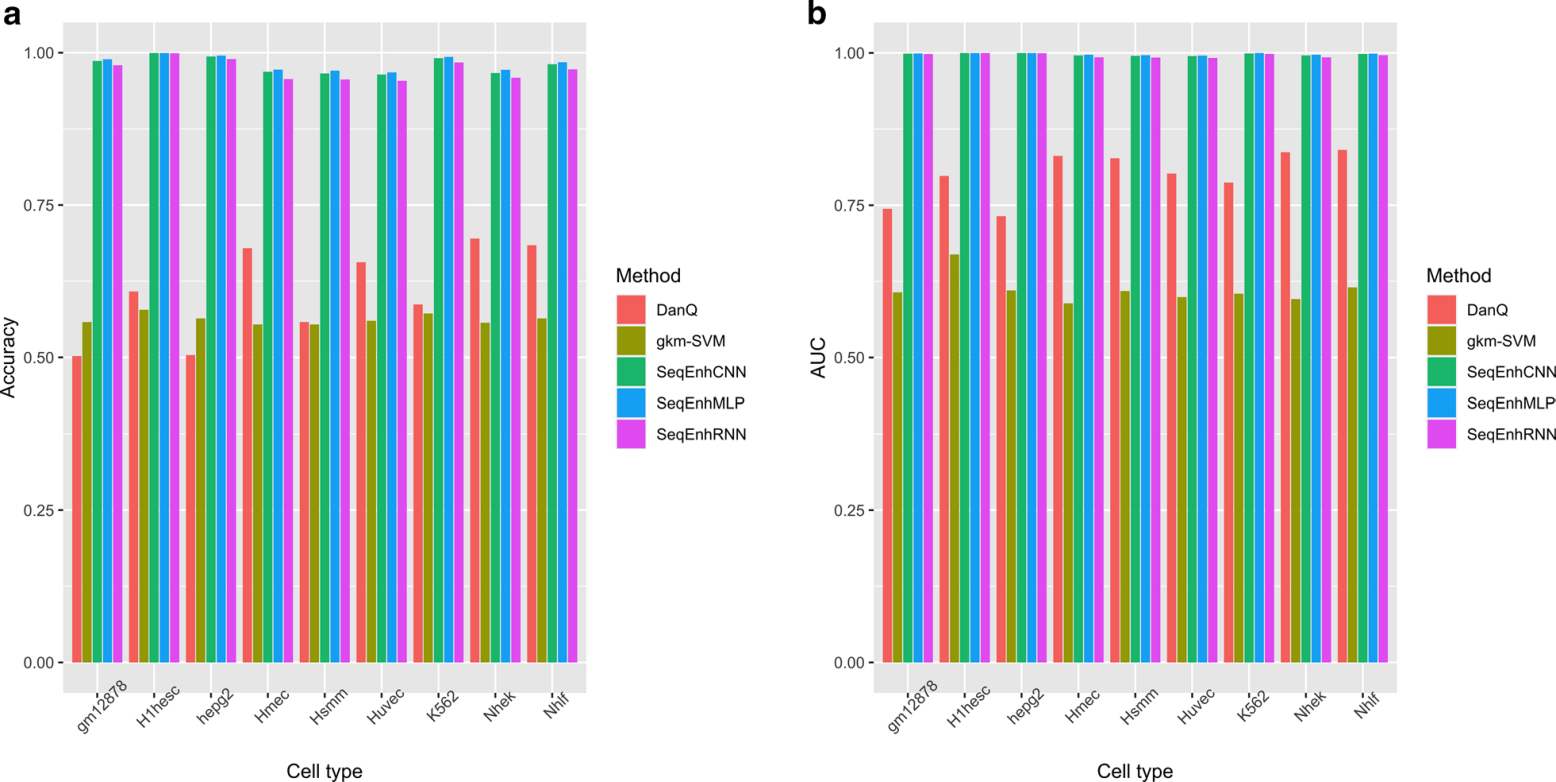
Comparison among different enhancer classifiers with regard to distinguishing cell type-specific enhancers from randomly selected non-coding sequences. **a** Comparison of accuracies. **b** Comparison of AUCs

Discriminating enhancers of a single cell type/tissue from randomly selected sequences have been studied before and provided the foundation for evaluating the performance of SeqEnhDL. We retrieved DNA sequences located within the “strong enhancers” chromatin states of nine cell types from the ENCODE project [14]. The performance of SeqEnhDL was evaluated in terms of accuracy and area under the curve (AUC) for distinguishing enhancers in each cell type. State-of-the-art methods were selected for comparison with SeqEhnDL. gkm-SVM [12, 17] was chosen for comparison because it uses *k*-mer information to predict enhancers. DanQ [28] was chosen for comparison because it is an RNN-based tool for predicting the functions of noncoding sequences. The performance of DanQ on each cell type was represented by the highest statistics among predictions on 919 ChIP/DNase-seq marks. When different tools were executed, five-fold cross-validation was employed in order to generate reliable performance measures. Comparisons of performances among different tools (Fig. 2) show that all the three models of SeqEnhDL greatly outperform gkm-SVM and DanQ. SeqEnhDL’s accuracies range from 0.961 to 0.999, suggesting that enhancers can be accurately identified on different cell types. Comparison of Receiver Operating Characteristic (ROC) curves on the hepg2 cell type (Additional file 1: Figure S2) re-confirmed that SeqEnhDL performed better. Of note, we also ran a recent approach based on the ensemble of deep RNNs [29] for a comparison. However, its accuracies and AUCs were around 0.5 (Additional file 1: Table S1), indicating that this compared approach was ineffective on this study’s datasets.

To validate the advantages of deep learning models over conventional machine learning models regarding enhancer classification, we flattened the *k*-mer features. We built enhancer classifiers based on six conventional machine learning models. Note that 2000 positive and negative sequences were randomly selected for each cell type and repeated ten times to ensure training each deep learning and conventional machine learning model could be finished within 1 h. Additional file 1: Figure S3 shows that accuracies of SeqEnhCNN and SeqEnhRNN are consistently higher than conventional machine learning models, and SeqEnhMLP is among the second tier in most cell types. These analyses collectively suggest that enhancers present in a single cell type can be accurately identified based on sequence features by SeqEnhDL, and SeqEnhDL significantly outperforms existing methods by better discriminating enhancers from randomly selected sequences.

### SeqEnhDL can discriminate enhancers’ cell types based on DNA sequences

**Fig. 3:**
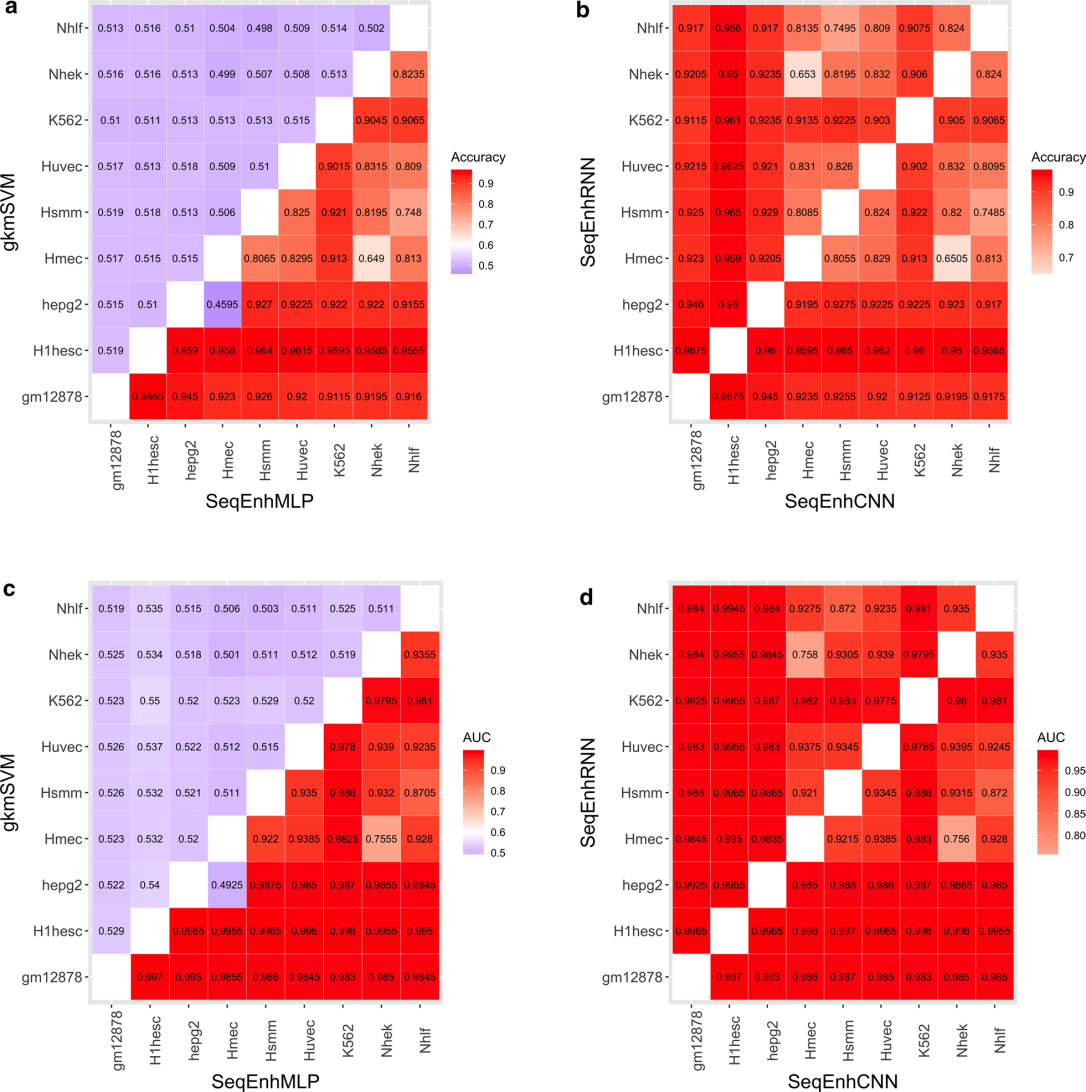
Comparison between gkm-SVM and SeqEnhDL with regard to discriminating enhancers from two cell types. **a**, **b** Comparison of accuracies. **c**, **d** Comparison of AUCs

Successful machine learning models for distinguishing enhancers from different cell types must learn cell-type-specific sequence structures such as domains, motifs, and their interactions. Previous enhancer classifiers were not examined regarding this capacity. Some may be adapted for distinguishing enhancers from different cell types by treating one cell type as the negative group. We applied gkm-SVM and SeqEnhDL to distinguish enhancers from different cell types. We switched the assignments of positive and negative groups for each pair of cell types and computed the average accuracy and AUC. The accuracies and AUCs for all pairs of cell types are displayed in Fig. 3. The accuracies and AUCs of gkm-SVM for all pairs of cell types are around 0.5, indicating that gkm-SVM failed to capture tissue-specificities of enhancers. In contrast, all models of SeqEnhDL generated high accuracies (e.g. > 0.9) and AUCs (e.g. > 0.95) in most cell type combinations, indicating that SeqEnhDL can identify tissue-specificity. This analysis suggests that SeqEnhDL can learn complex sequence features related to tissue-specificities and discriminate enhancers from different cell types.

## Discussion

When we built enhancer classifiers, we separated control and negative sequences. We used equal numbers of positive and negative sequences, which can significantly reduce the chances of overfitting, a common machine learning problem. We used all enhancers’ sequences to compute *k*-mer fold changes. Although theoretically, enhancers can be divided into two subsets (one for computing *k*-mer fold changes and the other for testing enhancer classifiers), it has practical limitations because longer *k*-mers are very important for composing enhancers and may occur only a few times in a cell type.

We successfully applied SeqEnhDL to discriminate enhancers from two cell types. gkm-SVM failed to distinguish enhancers from different cell types, indicating that most (if not all) previous *k*-mer based models tend to learn the common features of enhancers rather than tissue-specific motif structures. This successful application suggests that tissue/cell type-specific gene regulation could be better understood based on machine learning of high-level enhancers’ structures.

SeqEnhDL adopts positional *k*-mer fold changes as the sequence representation. This representation utilizes nucleotide positions, rather than *k*-mer indices. Positional *k*-mer fold changes reflect the degrees of k-mers’ specificity to a tissue/cell type regardless of the exact sequence. These positional *k*-mer fold changes increase the likelihood of capturing complex and high-level sequence features, which may aid the performance of SeqEnhDL.

Over a 200 bp sequence, important *k*-mers could appear at any position, and any important local patterns should be captured and contribute to enhancer prediction. Compared with conventional machine learning models, deep learning models extract high-level patterns from the features. Thus, it is not practical or reasonable to discriminate which nucleotide positions are more important than others when sequences are represented using positional *k*-mer fold changes.

## Conclusion

We propose SeqEnhDL, a feature extraction and deep learning framework for classifying cell-type-specific enhancers based on sequence features. A variety of analyses were performed to demonstrate that SeqEnhDL outperforms existing enhancer classifiers. We further proved that SeqEnhDL could be used to discriminate enhancers from different cell types.

## Limitation

The training dataset of this study from ChromHMM may be highly noisy. The primary goal of this study is to demonstrate the effectiveness of SeqEnhDL. SeqEnhDL is expected to perform better on cleaner datasets. Specific high-level features that are important for enhancer classification have not been addressed in this study and remain an open question.

## Supplementary Information

**Additional file 1:** **Table S1.** Accuracies and AUCs of the ensemble of deep RNN approach. ** Table S2.** Number of enhancers in each cell type after the filtering procedure. **Table S3.** Structures of deep learning models in SeqEnhDL. **Table S3.** Parameters of conventional machine learning models. ** Figure S1.** Flowchart of the detailed SeqEnhDL procedure. **Figure S2.** Comparison among different enhancer classifiers in terms of ROC curves.** Figure S3.** Comparison between SeqEnhDL and conventional machine learning models.

## Data Availability

Genome sequences and annotations were downloaded from UCSC genome browser (http://genome.ucsc.edu/). Chromatin state segmentation data were downloaded from the ENCODE project (http://hgdownload.soe.ucsc.edu/goldenPath/hg19/encodeDCC/wgEncodeBroadHmm/). Programs of this study were written in Perl, Python and R. All source code is freely available at https://github.com/wyp1125/SeqEnhDL. Testing datasets of SeqEnhDL for reproduction purpose are available at http://www.bdxconsult.com/SeqEnhDL. Supplementary methods, tables and figures are available in Additional file 1.
