# Supplementary material for: SeqEnhDL: sequence-based classification of cell type-specific enhancers using deep learning models
Source: BMC Res Notes. 2021 Mar 19;14:104. doi: 10.1186/s13104-021-05518-7 (PMC7980595; doi:10.1186/s13104-021-05518-7)
Supplement: Supplementary file 1 — Additional file 1: Table S1. Accuracies and AUCs of the ensemble of deep RNN approach. Table S2. Number of enhancers in each cell type after the filtering procedure. Table S3. Structures of deep learning models in SeqEnhDL. Table S3. Parameters of conventional machine learning models. Figure S1. Flowchart of the detailed SeqEnhDL procedure. Figure S2. Comparison among different enhancer classifiers in terms of ROC curves. Figure S3. Comparison between SeqEnhDL and conventional machine learning models. [file 13104_2021_5518_MOESM1_ESM.docx]

**Additional file 1**

**Methods**

**Feature extraction**

We masked exons and repetitive sequences of the human genome prior to retrieving DNA sequences for building enhancer classifiers. We divided all initial enhancers into 200bp enhancer units and treated each enhancer unit as an enhancer. 200bp units were utilized because the ENCODE chromatin state segmentation data was generated by the ChromHMM software which uses a 200bp scanning window. The number of enhancers in each cell type is shown in Table S2. Each enhancer was treated as a positive sequence. Both control and negative sequences were generated according to the GC contents of positive sequences. Control sequences, whose size was 3 folds the size of the positive sequences, were used to compute the background distributions of k-mers. Negative sequences, with the same size as the positive sequence set, were used as the negative set for training and testing enhancer classifiers. Thus, training data in this study was balanced with an equal number of positive and negative sequences. Fold changes of k-mer (k=5,7,9 and 11) frequencies (a pseudo count of 1 was added to both the denominator and numerator) were computed between the positive set and the control set and were used as feature dictionaries. Then, each 200bp sequence in the positive and negative sets was coded using the fold change of k-mers at each nucleotide position.

**Construction of machine learning models**

Positive sequences (with sizes ranging from 21,560 to 147,113) for any model were initially split into training and testing sets according to the 80:20 rule. Negative sequences were split according to the divisions of their corresponding positive sequences. For deep learning models, the initial training set was further divided into 70% training and 30% validation sets. Deep learning models were built based on the training and validation sets, and performance was assessed based on the testing set. Accuracies were defined as the proportion of correct classifications of the testing sequences. AUCs were computed based on the prediction scores on the testing sequences. Five-fold cross-validation was employed to generate reliable estimates of accuracies and AUCs (average from five runs).

**Deep learning models**

Deep learning models were generated using the Tensorflow and Keras software, available from Python3.7. Parameters were chosen heuristically and consistently to reach fair comparisons among different machine learning models and different cell types. Parameters included batch size: 512; learning rate: 0.001; epochs: 20; optimizer: Adam; loss: categorical_crossentropy. The best model during the 20 epochs was saved and used for prediction on the testing data. Structures of deep learning models are displayed in Table S3.

**Machine learning models involved in comparison**

gkm-SVM (1) was executed using default parameters. We also tested LS-GKM (2), which is the version of gkm-SVM used for large datasets, and found the two software generated very consistent outcomes. Fasta sequences, rather than *k*-mer features, were fed into gkm-SVM. Due to high computational burden of gkm-SVM, 2000 positive sequences and 2000 negative sequences were randomly selected from the original training datasets to train enhancer classifiers, while the entire dataset was used for testing.

DanQ (3) was executed using default parameters. Fasta sequences, which were extended to 1000bp from the 200bp bins, were fed to DanQ. DanQ made predictions on 919 ChIP/DNase-seq marks. For each cell type, the ChIP/DNase-seq mark with the highest accuracy was used to represent DanQ’s performance.

For Tan et al.’s enhancer classifier (4), original model structures and weights were downloaded and executed to classify the testing datasets of this study.

We note that the default parameters for competing methods might be sub-optimal on new datasets.

SVM models (linear and RBF kernels) and other conventional machine learning models including Decision Tree, Random Forest, AdaBoost, and Naïve Bayes were carried out using the scikit-learn python package. To ensure that computation was completed within one hour, 2000 positive sequences and negative sequences from the original training datasets were randomly selected for building conventional machine learning models. We found a sample size of 2000 sequences was enough to generate reliable and consistent estimates of accuracies for these conventional machine learning models. Parameter settings of conventional machine learning models are shown in supplementary Table S4.

**Computational resource**

All programs of this study were executed on the NIH Biowulf linux cluster. Tensorflow, Keras and required Python libraries were pre-configured under Python 3.7 on Biowulf. Deep learning jobs were executed on GPU nodes.

**References**

1. Ghandi, M., Lee, D., Mohammad-Noori, M. and Beer, M.A. (2014) Enhanced regulatory sequence prediction using gapped k-mer features. *PLoS computational biology*, **10**, e1003711.
2. Lee, D. (2016) LS-GKM: a new gkm-SVM for large-scale datasets. *Bioinformatics*, **32**, 2196-2198.
3. Quang, D. and Xie, X. (2016) DanQ: a hybrid convolutional and recurrent deep neural network for quantifying the function of DNA sequences. *Nucleic acids research*, **44**, e107.
4. Tan, K.K., Le, N.Q.K., Yeh, H.Y. and Chua, M.C.H. (2019) Ensemble of Deep Recurrent Neural Networks for Identifying Enhancers via Dinucleotide Physicochemical Properties. *Cells*, **8**.

**Tables**

**Table S1. Accuracies and AUCs of the ensemble of deep RNN approach**

| **Cell type** | **Accuracy** | **AUC** |
| --- | --- | --- |
| gm12878 | 0.522 | 0.529 |
| H1hesc | 0.530 | 0.544 |
| hepg2 | 0.524 | 0.532 |
| Hmec | 0.521 | 0.529 |
| Hsmm | 0.523 | 0.530 |
| Huvec | 0.522 | 0.530 |
| K562 | 0.521 | 0.530 |
| Nhek | 0.521 | 0.529 |
| Nhlf | 0.524 | 0.532 |

**Table S2. Number of enhancers in each cell type after the filtering procedure**

| **Cell type** | **Number of enhancers** |
| --- | --- |
|  |  |
| gm12878 | 92,763 |
| H1hesc | 21,560 |
| hepg2 | 69,337 |
| Hmec | 136,641 |
| Hsmm | 145,952 |
| Huvec | 147,113 |
| K562 | 80,912 |
| Nhek | 136,803 |
| Nhlf | 111,889 |

**Table S3. Structures of deep learning models in SeqEnhDL**

| **Deep learning model** | **Structure** |
| --- | --- |
| MLP | Dense(400, activation='relu')  Dropout(0.5)  Dense(400, activation='relu')  Dropout(0.5)  Flatten()  Dense(2, activation='softmax') |
| CNN | Conv2D(32, kernel_size=(4, 1), strides=(1, 1), activation='relu')  MaxPooling2D(pool_size=(4, 1), strides=(4, 1))  Conv2D(64, kernel_size=(5, 1), strides=(1, 1),activation='relu')  MaxPooling2D(pool_size=(5, 1), strides=(5, 1))  Flatten()  Dense(400, activation='relu')  Dropout(0.5)  Dense(2, activation='softmax') |
| RNN | Bidirectional(layers.LSTM(128))  Flatten()  Dense(128, activation='relu')  Dropout(0.5)  Dense(2, activation='softmax') |

**Table S4. Parameters of conventional machine learning models**

| **Machine learning model** | **Parameters** |
| --- | --- |
| Linear SVM | C=1 |
| RBF SVM | gamma=0.01, C=2 |
| Decision Tree | max_depth=5 |
| Random Forest | Max_depth=5, n_estimators=10, max_features=1 |
| AdaBoost | Default |
| Naïve Bayes | Default |

**Figures**

**
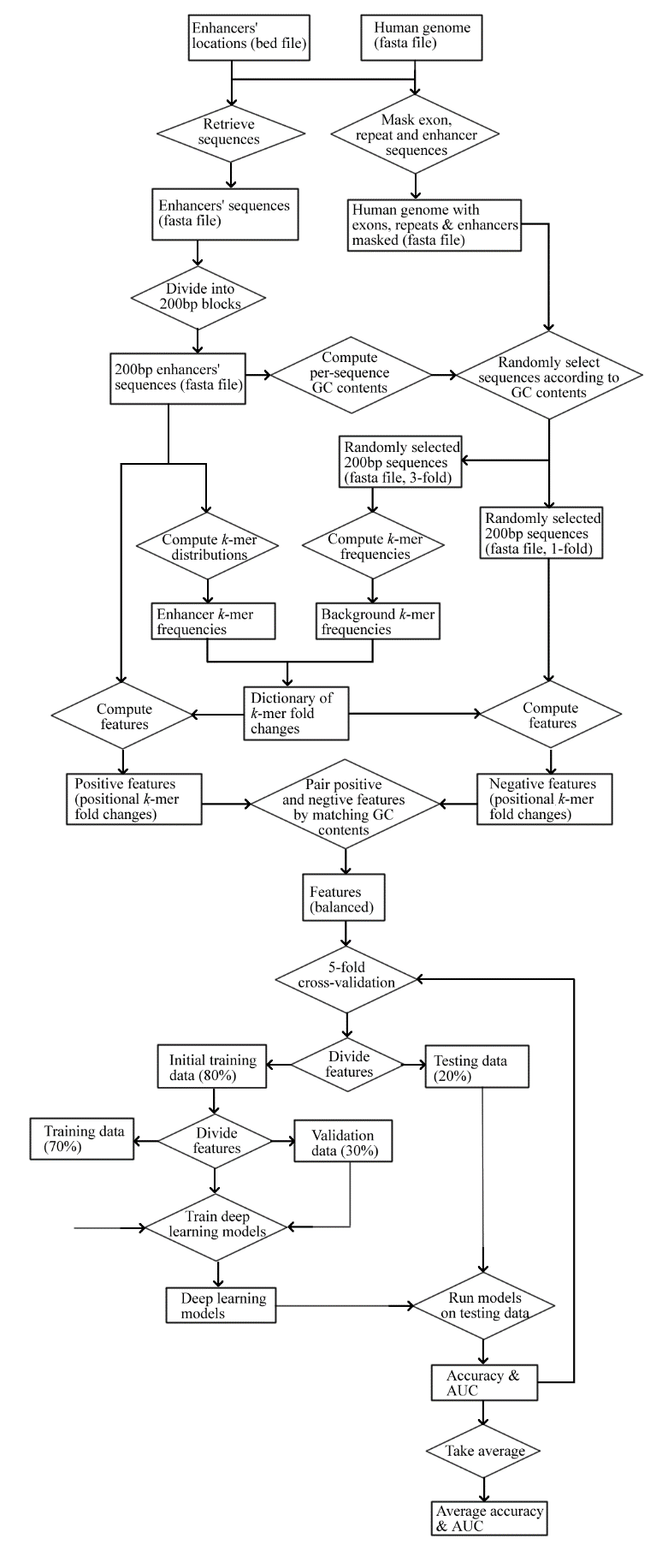
**

**Figure S1. Flowchart of the detailed SeqEnhDL procedure.**

**
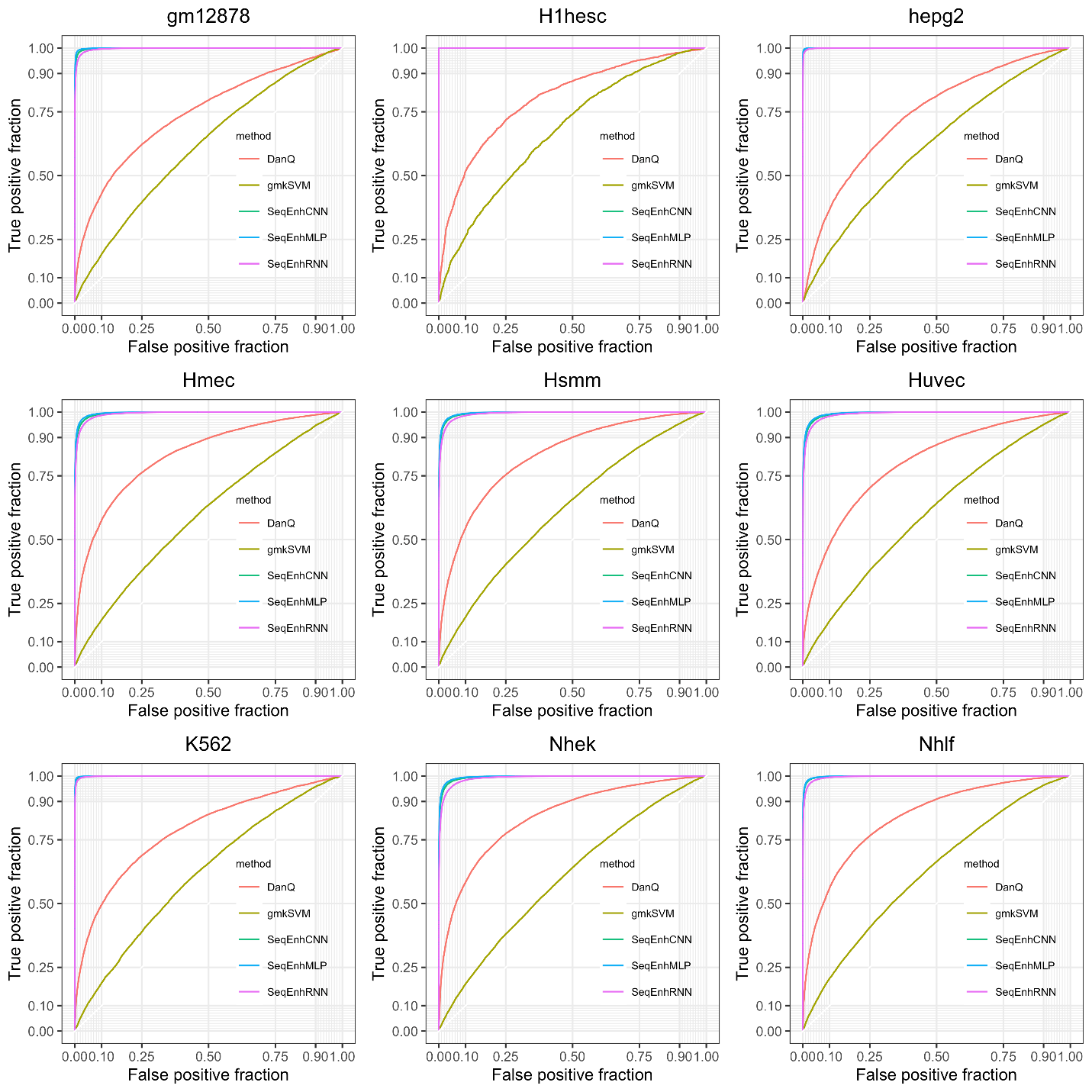
**

**Figure S2. Comparison among different enhancer classifiers in terms of ROC curves**. The curves were generated based on the first cross-validation dataset of each cell type.


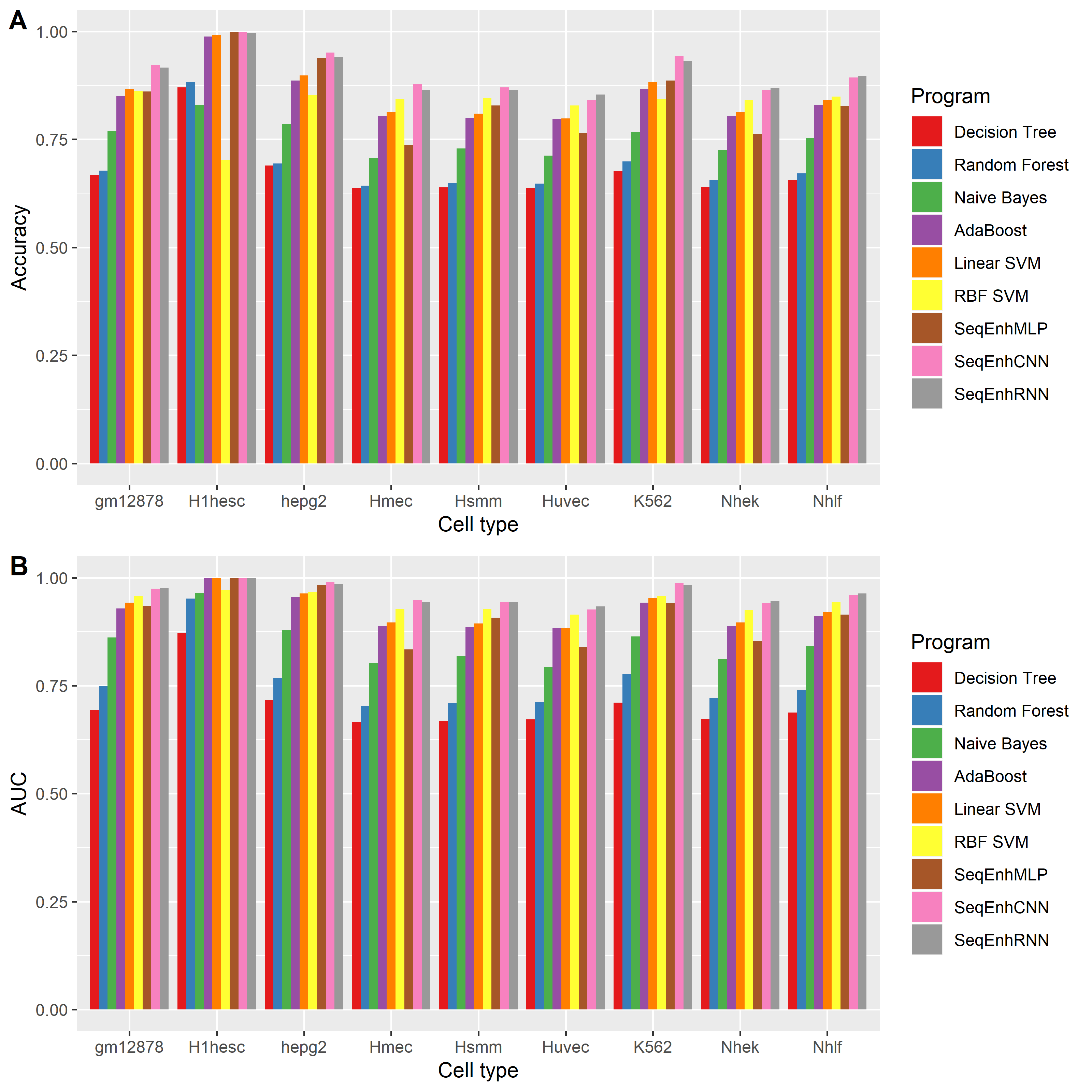


**Figure S3. Comparison between SeqEnhDL and conventional machine learning models. (**A) Comparison of accuracies. (B) Comparison of AUCs.
